# Evaluation of the effects of a gel containing green tea and hyaluronic acid on clinical and Microbiological outcomes following periodontal surgery. A randomized, controlled, double-blinded, noninferiority trial

**DOI:** 10.1007/s00784-026-06792-0

**Published:** 2026-02-24

**Authors:** Laura Sanches Gonçalves, Stéffany Souza Melo, Guilherme José Pimentel Lopes de Oliveira, Luciene Cristina Figueiredo, Fabio José Barbosa Bezerra, Flávia Aparecida Chaves Furlaneto, Sérgio Luís Scombatti de Souza

**Affiliations:** 1 Department of Oral and Maxillofacial Surgery and Periodontology, School of Dentistry of Ribeirao Preto, University of Sao Paulo, Av do Café, s/n., Ribeirao Preto, SP 14040-904 Brazil; 2 Department of Periodontology/Implantodontology, School of Dentistry, Universidade Federal de Uberlândia - UFU, Uberlândia, Brazil; 3 Department of Periodontology, School of Dentistry, Guarulhos University, São Paulo, Brazil

**Keywords:** Periodontitis, Wound healing, Oral surgical procedures, Chlorhexidine, Green tea, Hyaluronic acid

## Abstract

**Objectives:**

To evaluate the clinical and microbiological effects of a gel containing green tea and hyaluronic acid (GT/HA) following open-flap debridement surgery in comparison with those of a chlorhexidine-based gel.

**Materials and methods:**

This randomized, controlled, double-blind, noninferiority trial included 38 patients with stage 3 periodontitis. After undergoing surgery, participants were randomly assigned to receive either GT/HA gel or chlorhexidine gel three times daily for 14 days. Clinical parameters (plaque index, probing depth, bleeding on probing, and clinical attachment level) were evaluated at baseline and 60 days postoperatively. Subgingival biofilm samples were collected at baseline and after 14 and 30 days and analyzed using checkerboard DNA–DNA hybridization for 40 bacterial species. Patient-reported outcomes included postoperative pain, analgesic consumption, and quality of life assessed by the OHIP-14 questionnaire.

**Results:**

Both groups showed statistically significant clinical improvements from baseline to 60 days, with no significant differences between treatments. Temporary reductions in some bacterial species were observed during the 14-day gel application period in both groups, followed by regrowth after discontinuation. GT/HA gel was associated with fewer adverse effects, particularly regarding taste alteration and tooth or tissue staining. A reduction in OHIP-14 scores was observed in both groups, with statistically significant differences between baseline and 30 days after surgery.

**Conclusions:**

GT/HA gel was noninferior to chlorhexidine gel in terms of clinical and microbiological outcomes and showed greater patient tolerability.

**Clinical relevance:**

GT/HA gel may represent a safe and effective alternative to chlorhexidine in postoperative care in periodontal surgery.

## Introduction

Periodontitis is a multifactorial chronic inflammatory disease that is characterized by progressive destruction of the tooth support apparatus caused by excessive immune responses and microbiota dysbiosis [1–3]. Clinically, periodontitis manifests as bleeding on probing, loss of clinical attachment, bone loss, and tooth mobility and can progress to tooth loss if left untreated [3].

After periodontitis has been diagnosed, an appropriate treatment protocol must be instituted considering the stage and degree of the disease to achieve the following objectives: stopping the progression of the disease, removing biofilm and subgingival calculus, improving clinical parameters, and reducing tissue inflammation [4]. After cause-related therapy, patients who do not achieve adequate clinical improvement may require additional treatment, including reinstrumentation of persistent sites or a surgical approach to control disease activity [5].

After surgical therapy, the patient is generally instructed not to brush the teeth involved so that no trauma to the surgical area occurs [6]; furthermore, the intervention site can become more sensitive, causing discomfort to patients during mechanical cleaning procedures, which decreases the cleanliness of regions close to the surgical wound [7]. Therefore, the dentist must use adjuvant agents during this postoperative period, since adequate biofilm control (chemical control) will allow better and faster tissue healing, prevent local infections and, therefore, guarantee the success of the procedure [6, 8, 9].

Among the antimicrobial agents used for chemical biofilm control, chlorhexidine is considered the gold standard. Although it has great properties, such as substantivity, which gives it a prolonged action time, and is effective, it can promote microbial resistance [6], and adverse effects of chlorhexidine have been reported in the literature, mainly during prolonged use, such as pigmentation of teeth and other oral tissues, irritation of the oral mucosa, changes in taste, cytotoxic effects on human gingival fibroblasts, and the condition of burning mouth [10–12]. Given this, some authors have tested other agents to be incorporated into toothpastes or mouthwashes, especially when long-term use is intended, such as herbal medicines, which do not exert the adverse effects of chlorhexidine and have antimicrobial and anti-inflammatory properties [13, 14].

An herbal medicine widely studied in the literature is green tea extract, which is obtained from the leaves of the *Camellia sinensis* plant. During the process of obtaining it, polyphenols and catechins, which are attributed to the health benefits of green tea, are preserved [15]. Some properties of green tea include antibiofilm effects, inhibitory effects on gingival inflammation, and antibacterial and antioxidant effects [15, 16]. In a randomized clinical trial, Hrishi et al. (2016) reported that the use of a green tea-based toothpaste resulted in reduced inflammation and increased clinical attachment in patients with periodontal disease, and these findings were associated with the antioxidant effect of green tea [17].

In addition to antimicrobial effects from mouthwashes or gels used during the postoperative period, substances that stimulate healing are also desirable for promoting the success of a surgical procedure. Hyaluronic acid is a constituent of connective tissue and plays an important role in tissue regeneration, facilitating cell migration and differentiation during tissue formation and repair [18]. Mamajiwala et al. (2021) [19] reported a greater increase in clinical attachment and a greater reduction in probing depth in a treatment group in which a hyaluronic acid gel (0.8%) was administered during the postoperative period of periodontal surgery than in the control group. In another study, mouthwash supplemented with hyaluronic acid reduced inflammation in patients with gingivitis to the same degree as chlorhexidine had [20].

A new gel containing these two components, green tea extract and hyaluronic acid, was evaluated after third molar extraction. A relevant finding in this study was the result of human fibroblast culture, which revealed a synergistic effect of these two gel components on the upregulation of the expression of the AKT gene, a key indicator of cell survival. When combined, the compounds led to an approximately 100-fold increase in AKT gene expression compared with that in the control group, revealing the important therapeutic potential of this product for healing and tissue regeneration. Furthermore, in the clinic, this gel favors the healing of soft tissues and bones after extraction, offering a promising adjuvant therapy to improve postoperative recovery [21].

The combination of green tea and hyaluronic acid may provide complementary biological effects that are particularly beneficial after periodontal surgery. Green tea has antimicrobial, antibiofilm, and anti-inflammatory properties, while hyaluronic acid plays important roles in cell migration and tissue repair. Their combined use could therefore help control biofilm formation and local inflammation, creating a favorable environment for postoperative tissue recovery [15–19].

Thus, considering the need to control biofilm formation and stimulate tissue healing after surgical therapy in periodontitis patients and considering the beneficial effects demonstrated in the literature of compounds using green tea or hyaluronic acid, the objective of this study was to evaluate the effects of a new gel containing these two associated substances administered to patients during the postsurgery period after open flap debridement surgery and to compare its effects on tissue repair and microbial control in relation to those of a chlorhexidine-based control gel.

## Objectives

### General Objective

To evaluate the clinical and microbiological effects of a gel containing green tea and hyaluronic acid (GT/HA) following open-flap debridement surgery in comparison with those of a chlorhexidine-based gel.

### Specific Objectives

1. To evaluate the influence of a gel containing green tea extract and hyaluronic acid on clinical outcomes, specifically the plaque index (PI), probing depth (PD), bleeding on probing (BOP), and clinical attachment level (CAL);
2. To evaluate the microbial control performance of the gels applied during the postoperative period using the checkerboard DNA–DNA hybridization technique;
3. The outcome measures reported by patients were evaluated through an analysis of postoperative painful symptoms using a visual analog scale (VAS), the consumption of analgesics used during the period, responses to an oral health impact profile questionnaire (OHIP-14) and a postoperative signs and symptoms questionnaire, and an analysis of discomfort, edema, bleeding and tooth sensitivity also using a visual analog scale (VAS).

## Materials and methods

### Study design

This study was a parallel, double-blind, randomized clinical trial. A total of 38 patients were selected from the population referred for periodontal treatment at the Faculty of Dentistry of Ribeirao Preto (FORP). All eligible subjects signed the informed consent form. The study protocol was evaluated and approved by the institution’s Human Research Ethics Committee (protocol number 60530022.1.0000.5419). Furthermore, the study was registered in the Brazilian Clinical Trials Registry (ReBEC - number RBR-475mc4q).

### Sample size calculation

For statistical purposes, the patients were considered individual cases, and clinical attachment level (CAL) was the primary outcome variable. The sample size calculation was performed on the basis of clinical attachment level data from the study by Cadore et al. [22] after surgical therapy for the treatment of periodontal disease. After surgical therapy, the groups presented mean and standard deviation values of 5.59 ± 0.54 mm and 5.03 ± 0.36 mm, respectively (Cadore et al., 2018), and a β power of 0.95 and an α power of 0.05 were used; thus, 16 patients per group was determined to be sufficient to carry out this study with adequate statistical power. Considering a dropout rate of 20%, 19 patients were selected per group for the present study. Although the reference study reported outcomes at 3 months, the use of its data for sample size estimation was considered appropriate since the calculation was based on the expected difference between groups rather than the time of evaluation. In accordance with the noninferiority design of the present study, a noninferiority margin (Δ) of 0.5 mm in the CAL was adopted, as differences below this threshold are considered clinically irrelevant in clinical periodontal trials. Statistical analyses were performed considering a one-sided α of 0.05 to test for noninferiority.

### Sample characterization

The inclusion criteria were patients with stage 3 periodontitis that completed periodontal therapy related to the cause more than one month ago and still had sites with two or more adjacent teeth presenting pockets with a probing depth ≥ 5 mm and bleeding on probing and/or suppuration, in addition to good oral hygiene standards (BoP ≤ 20% and PI ≤ 20% [23]).

The exclusion criteria were patients who received antibiotic treatment in the previous 6 months; patients with systemic conditions that could interfere with postsurgical repair or progression of periodontal disease, such as diabetes; patients taking medications that affect the gums and/or oral mucosa (phenytoin and other antiepileptic agents; cyclosporine; calcium channel blocking agents); patients who smoked; patients who were pregnant; and patients with a history of allergy to any of the components that were used in the present study.

Patient recruitment took place from January 2023 to October 2023.

### Examiner calibration

All clinical parameters were measured by a single calibrated examiner (L.S.G.). In two separate sessions 15 days apart, duplicate measurements of PD and CAL were obtained from eight patients who were not included in this study. For quantitative variables, analysis was performed using the intraclass correlation coefficient (ICC), which was greater than 0.75.

### Sample randomization

Patients were randomized into two study groups (chlorhexidine/*n* = 19 and GT/HA/*n* = 19) using a random sequence generated by Microsoft Excel Software (Microsoft Inc., Redmond, WA, USA). Each patient was assigned a unique identification number (0–38) in an Excel spreadsheet prepared by an independent assistant who was not involved in the clinical procedures or data analysis. This procedure ensured random allocation and preserved patient confidentiality during data recording and statistical analysis.

### Study blinding

This study was conducted in a double-blind manner. The test and control gels were identically packaged and labeled with neutral codes (“1” or “2”) by a third party not associated with the study or the sponsoring company. Neither the patients nor the examiner/operator knew the group allocation during the study. The statistician remained blinded to the treatment groups until all the data analyses were completed. These procedures ensured full masking of group allocation and prevented potential bias related to the authors’ affiliations.

### Clinical assessment

Patients were clinically evaluated at baseline (T0), and after 2 months of surgery (T60), all the parameters were measured by a single trained examiner who was blinded to the treatments. Periodontal measurements were performed using a UNC-15 probe (University of North Carolina probe, Hu-Friedy, Chicago, IL). The following measurements were taken only on the teeth involved in the surgery:

- Probing depth (PD): measured on 6 dental surfaces (mesiobuccal, center of the buccal face, distobuccal, mesiolingual/palatal, center of the lingual/palatal face, distolingual/palatal) with a North Carolina millimeter probe and defined as the distance from the gingival margin to the bottom of the sulcus or pocket.
- Bleeding on probing (BOP): BOP was measured as the occurrence of bleeding on gentle probing up to 15 s after the probe was removed from the sulcus, and the sites where this bleeding occurred were noted on the periogram, as proposed by Ainamo; Bay (1975) [24]. This evaluation was also carried out on six surfaces per tooth: mesiobuccal, center of the buccal face, distobuccal, mesiolingual/palatal, center of the lingual/palatal face, and distolingual/palatal.
- Clinical attachment level (CAL): This measurement was taken on six surfaces per tooth: mesio-buccal, center of the buccal face, disto-vestibular, mesio-lingual/palatal, center of the lingual/palatal face, and disto-lingual/palatal. This measurement was obtained using a North Carolina millimeter probe, determined by the distance from the cementoenamel junction to the bottom of the groove or pocket.
- Plaque index (PI): This index was determined according to the methods previously described in O’leary; Drake; Naylor (1972) [25]. The teeth present in the oral cavity were divided into four surfaces: buccal, palatal/lingual, mesial and distal. The number of faces containing plaque was multiplied by 100, and the value obtained was divided by multiplying the number of teeth present by four.

### Intervention

The selected patients were randomly divided into two groups.

- Control Group (chlorhexidine-based gel): Patients who received a chlorhexidine-based gel at the intervention site after periodontal surgery.
- Test group (new gel): Patients who received a gel containing green tea extract and hyaluronic acid at the intervention site after periodontal surgery.

### Gel composition and characteristics

The gel used in this study contained the following components (New Dental Care, Ribeirao Preto, Sao Paulo, Brazil): 1.00% carboxymethylcellulose, 10.00% glycerin, 60.00% sorbitol, 0.30% sodium benzoate, 0.50% xylitol, 0.50% laurel glucoside, 0.05% hyaluronicpyrrolidone K 30, 0.50% dimethylsilanediol salicylate (DSBC), 10.00% thixosil 43B, 5.00% thixosil, 0.5% tetrasodium pyrophosphate, 0.50% saccharin, 0.50% green tea extract (C*amellia sinensis*), 2.00% hydrogenated castor oil, 0.05% EDTA, and 11.45% purified water [21]. 

The test gel was transparent and presented a low viscosity similar to that of the control gel. The control gel contained chlorhexidine at a concentration of 0.2%.

### Surgical procedures

**Fig. 1:**
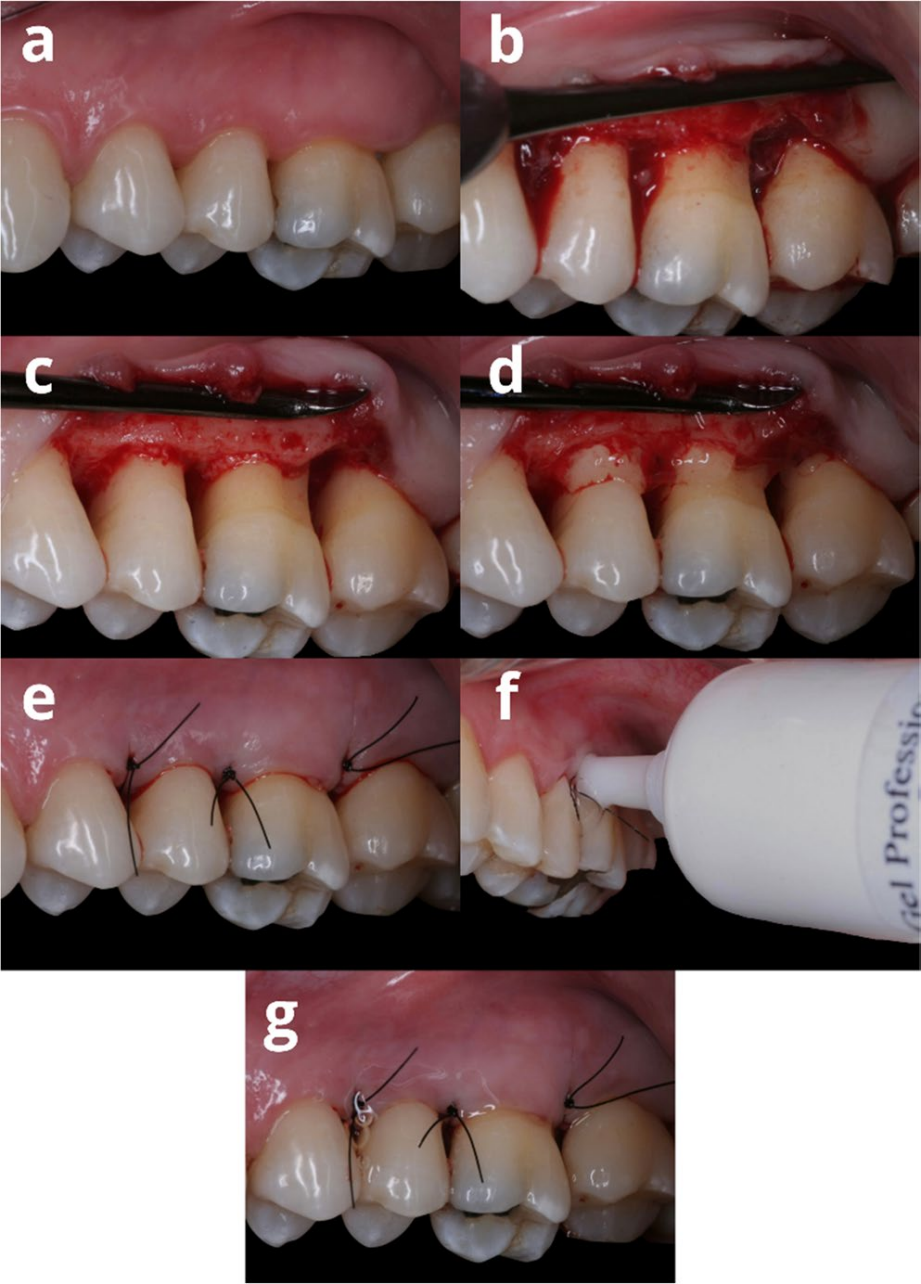
Surgical sequence and gel application. (**a**) Clinical situation prior to surgery. (**b**) Flap prepared and detached. (**c**) Root surfaces after mechanical debridement. (**d**) Application of EDTA for chemical decontamination of root surfaces. (**e**) Sutures performed. (**f**) Application of the gel. (**g**) Clinical aspect of the immediate postoperative period and with gel applied to the dental elements involved in the surgery

During the baseline period, surgery was performed by a single previously trained operator (L.S.G.). For the procedure, surgery was performed at sites with a PD ≥ 5 mm and the presence of a BoP. After local anesthesia, an intrasulcular incision with a 15 C scalpel blade (Swann-Morton^®^) was performed, encompassing the site with a PD ≥ 5 mm and preserving the gingival papillae. A mucoperiosteal flap was then raised until bone crest exposure, and subgingival calculus and/or granulation tissue deposits were removed with conventional Gracey and Mini-Five curettes (Hu-Friedy, Chicago, IL, USA), ultrasonic devices and drills if necessary to smooth the tooth surface. After the root surface was mechanically treated, a 24% EDTA (ethylenediaminetetraacetic acid) solution in the gel at neutral pH was applied for 2 min, followed by abundant irrigation with saline for 30 s. Finally, interproximal sutures (nylon 5 − 0) were used for flap repositioning (Fig. 1).

At the end of surgery, the assistant checked on a spreadsheet that the operator did not have access to which gel number the patient should receive, and then the operator applied the gel to the patient and carried out standardized postoperative hygiene care instructions. Before applying the gel, the region was first dried with gauze to prevent saliva from removing the gel from its position. Then, the applicator tip of the packaging was used to apply the gel around the gingival tissues of the dental elements involved in the surgery on the buccal and lingual sides. Patients were instructed not to ingest liquids or food for 30 min after application.

Each patient subsequently received a tube of gel corresponding to the group in which they were allocated so that they could apply the gel three times a day for up to fourteen days after surgery. Furthermore, after the procedure, each patient was given a specific brush for postsurgical care (Soft Brush, IMPLANTS line, New Dental Care) and a toothpaste (developed by the company New Dental Care without active ingredients, so as not to interfere in research analyses) to standardize the oral hygiene habits of these patients. The application of the gels was monitored through the diary and the return of the tubes at the end of the application.

### Postoperative diary and questionnaire

Immediately after surgery, patients received a diary to record their pain intensity on a visual analog scale (VAS) for 14 days, as well as the dose of analgesics ingested during the postoperative period and the number of daily applications of the gel. All patients received the same medication, which consisted of an analgesic (Dipyrone Sodium 500 mg, 6/6 hours, if necessary), for 3 days.

Furthermore, during follow-up seven, fourteen and thirty days after surgery, patients were subjected to a postoperative Signs and Symptoms Questionnaire using a visual analog scale (VAS), in which the patient responded to questions regarding discomfort, bleeding, edema and other complications after surgery.

### Appointments for postoperative follow-up

**Fig. 2:**
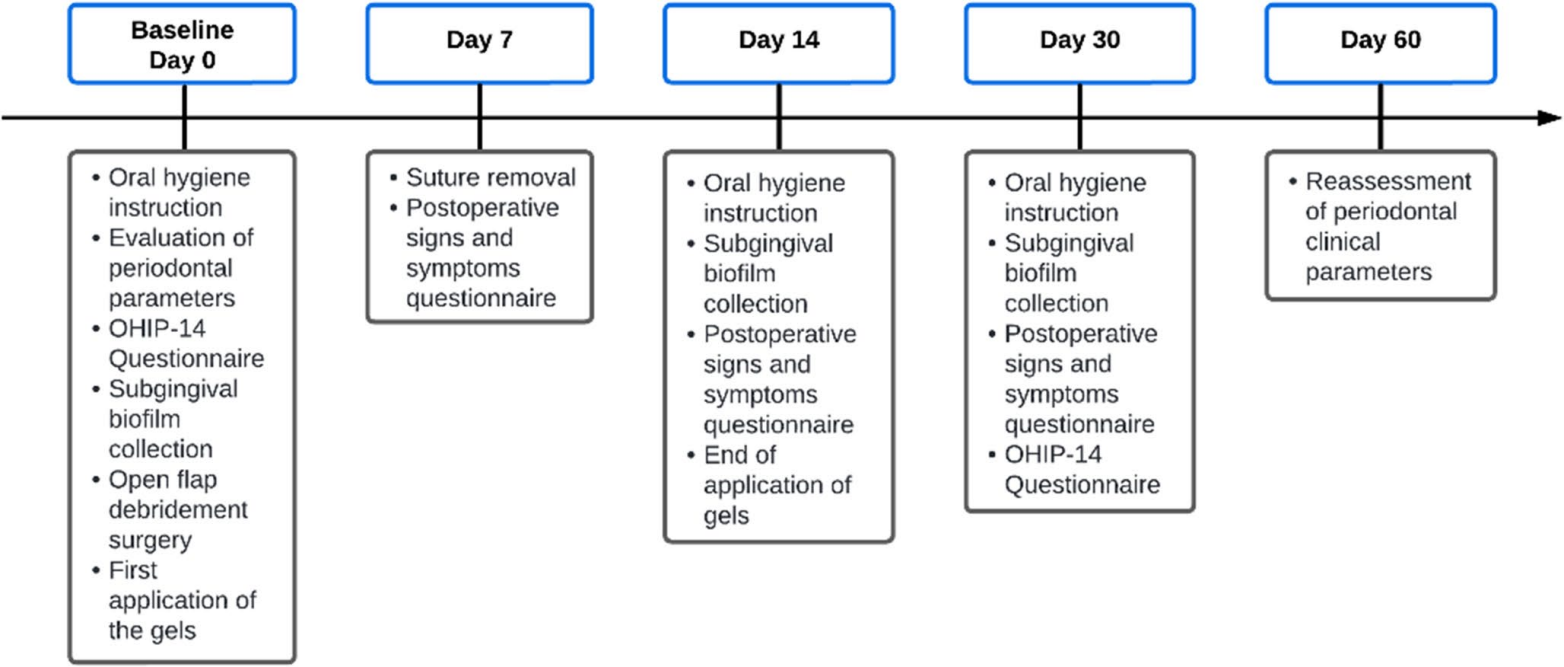
Study timeline

The study sessions took place as illustrated in the timeline below (Fig. 2).

One week after surgery, the patient returned to the School of Dentistry of Ribeirao Preto to have their sutures removed.

Fourteen days after surgery, reinforced oral hygiene instructions were given to patients, and biofilm collection was carried out again. These same procedures were performed upon return at 30 days after surgery.

Finally, 60 days after surgery, patients returned for reassessment of clinical parameters such as the plaque index, (PI), probing depth, (PD), bleeding on probing, (BOP) and clinical attachment level, (CAL)

### Oral health impact profile questionnaire

Oral health-related quality of life (OHRQoL) was measured using the Oral Health Impact Profile (OHIP-14) questionnaire, a subjective tool that aims to provide a measure of disability, discomfort, and disadvantage attributed to oral condition through self-assessment. The questions are scored on a Likert scale (0 indicates never; 1, rarely; 2, sometimes; 3, constantly; and 4, always). To obtain the final score of the scale, which can range from 0 to 56, the score of each item was added. The higher the score is, the worse the OHRQoL. To verify patients’ experience with the treatments instituted, patients completed this questionnaire at two time points: before surgery and 30 days after the procedure. For the present study, a validated version of the OHIP-14 questionnaire translated into Brazilian Portuguese was used [26].

### Microbiological monitoring

Samples of the subgingival microbiota present in the periodontal pockets were collected at three different times: baseline (immediately before surgery), 14 days after surgery and 30 days after surgery. The material was collected only from sites (PD > 5 mm and BoP) of the teeth involved in surgery, and a biofilm pool of the patient was obtained. The number of pooled sites per patient varied depending on the number of pockets with probing depths between 5 and 7 mm, with approximately 3 to 4 sites per patient. For this collection, the selected teeth were isolated with sterile cotton rolls and dried with air jets, avoiding contamination of the area with salivary fluid. With the aid of a sterile Gracey curette (Gracey Curettes, Hu-Friedy, Chicago, IL, USA), the supragingival biofilm was carefully removed. Another sterile Gracey curette (mini) was subsequently used to collect the subgingival microbiota from the mesial and distal sites, starting from the deepest portion of the pocket in the coronal direction of each selected tooth element. Immediately after collection, the sample from each site was stored in a specific sterile Eppendorf-type bottle containing 150 µL of buffer solution (10 mM Tris-HCL, 1 mM EDTA, pH 7.6 – Tris-EDTA (TE) solution). One hundred microliters of 0.5 M sodium hydroxide (NaOH) was added to this solution so that the bacterial deoxyribonucleic acid (DNA) remained viable for a long period of time. The Eppendorfs, duly identified with the patient code, date and site collected, were stored under refrigeration at -80 °C. At the Dental Research Laboratory II of the University of Guarulhos (University of Guarulhos – UNG, Guarulhos, SP, Brazil), the checkerboard DNA–DNA hybridization microbial analysis method was used to count 40 subgingival bacterial species in each sample [27, 28].

### Statistical analysis

All the statistical analyses were performed using GraphPad Prism 9 (San Diego, CA, USA), with a significance level of 5%.

The normality of the clinical data was assessed using the Shapiro–Wilk test, and the homogeneity of variance across groups was verified. Given that the variances were normally distributed and that the parametric assumptions were met, two-way ANOVA followed by Tukey’s post hoc test was used to evaluate the interaction between the independent variables, treatment and evaluation time. For outcomes with a single measurement per time point (e.g., number of analgesics consumed, average amount of gel applied during the experimental period), the paired t test was employed.

For microbiological parameters, nonparametric tests were applied. The Mann–Whitney U test was used to compare groups at different experimental periods, while the Wilcoxon signed-rank test was used to compare different periods within each treatment group.

## Results

**Fig. 3:**
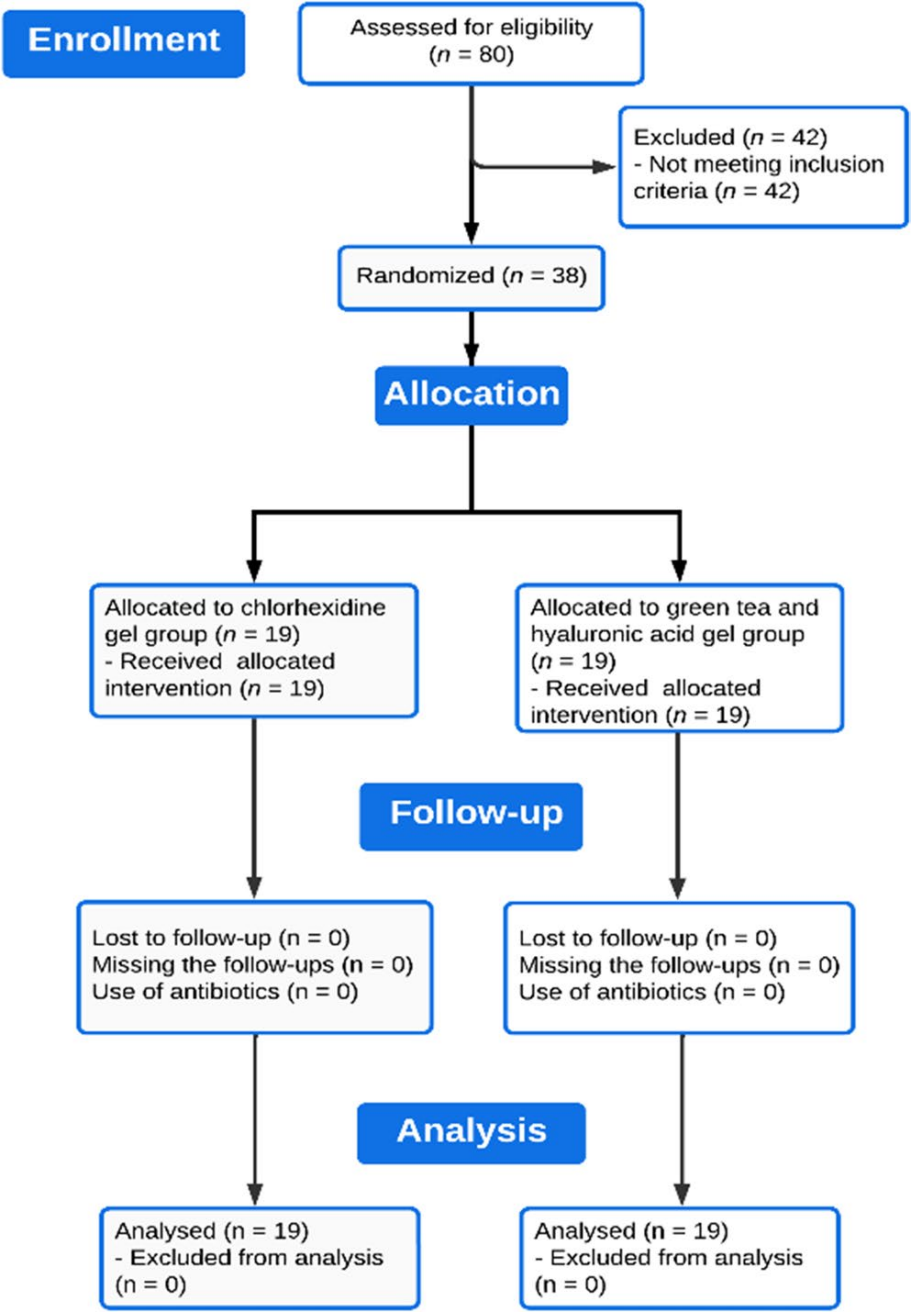
Study flowchart

The flow chart of the study design is shown in Fig. 3. All the subjects successfully completed the study.

### Clinical outcomes

**Fig. 4:**
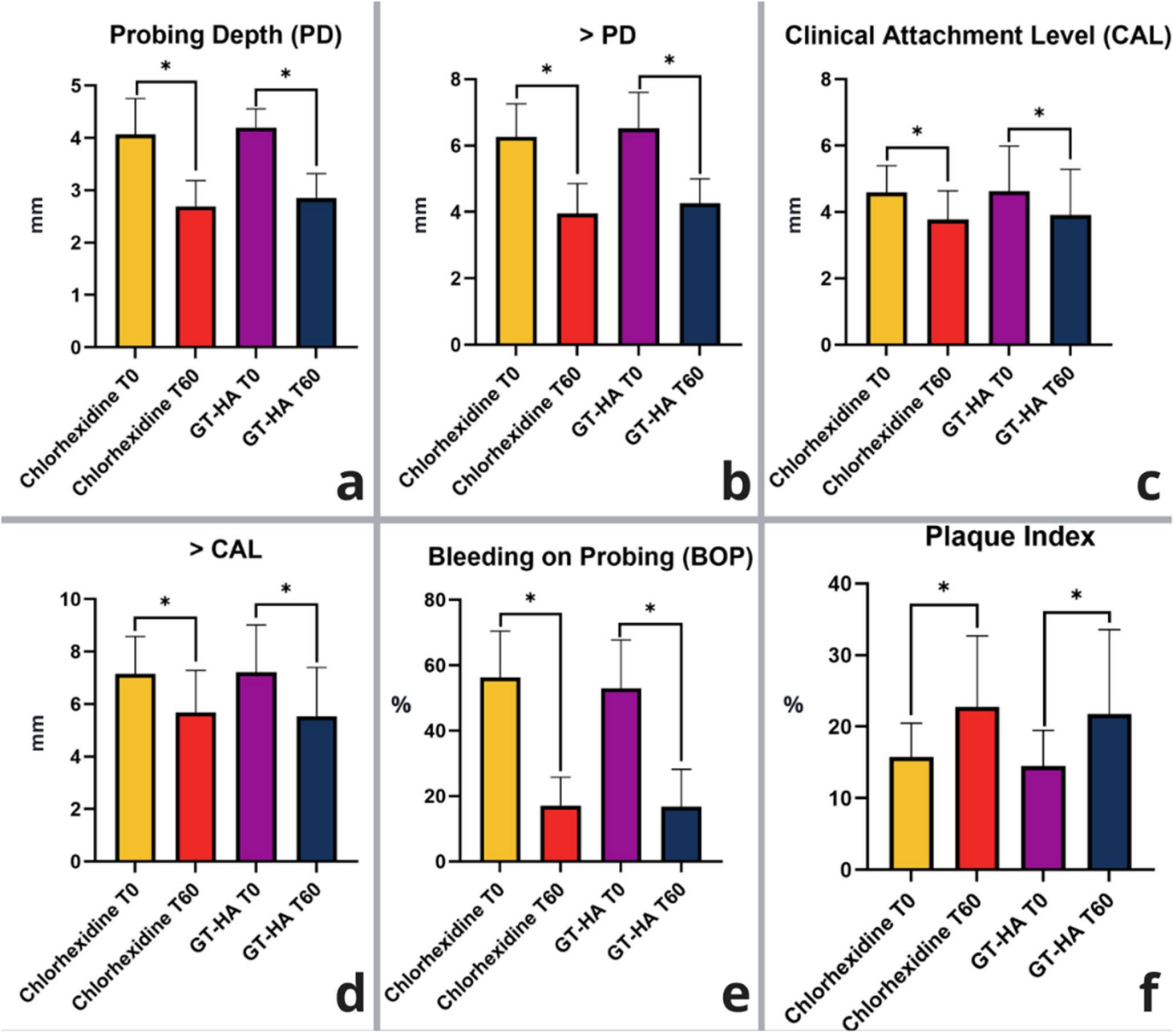
Clinical outcomes. (**a**) Probing Depth; (**b**) > Probing Depth; (**c**) Clinical Attachment Level; (**d**) > Clinical Attachment Level; (**e**) Bleeding on Probing Index; (**f**) Plaque Index. PD = probing depth; CAL = clinical attachment level; * = statistically significant difference (*p* < 0.05 - two-way ANOVA complemented by Tukey’s test); GT = green tea; HA = hyaluronic acid

The results of the present study demonstrate that the use of a gel containing green tea and hyaluronic acid during the postoperative period after periodontal surgery is non-inferior to the use of a chlorhexidine-based gel. With respect to the probing depth (PD) parameter, it decreased from 4.06 ± 0.68 mm to 2.69 ± 0.48 mm in the chlorhexidine-based gel group (*p* < 0.0001) and from 4.19 ± 0.35 mm to 2.85 ± 0.47 mm (*p* < 0.0001) in the group in which the green tea and hyaluronic acid-based gel was used. With respect to the clinical attachment level (CAL), reductions from 4.59 ± 0.80 mm to 3.77 ± 0.85 mm (*p* < 0.0001) were also observed in the chlorhexidine-based gel group and from 4.62 ± 1.35 mm to 3.90 ± 1.38 mm (*p* < 0.0001) in the green tea and hyaluronic acid-based gel group. An increase in the plaque index (PI) was observed for both groups at the end of the study. For the CHX group, the increase ranged from 15.74 ± 4.74% to 22.74 ± 9.96%, and for the GT/HA group, it ranged from 14.53 ± 4.95% to 21.79 ± 11.76% (*p* < 0.0001) (Fig. 4). There were no statistically significant differences between the two treatment groups with respect to PD and BoP reduction or CAL gain. Both treatments were successful and nearly identical for clinical measures, with both groups demonstrating a significant difference between the presurgery and 2-month posttreatment time points (Table 1).

**Table 1:** Mean and standard deviation data of the variation in clinical parameters between T0 and T60. Two-way ANOVA complemented by tukey’s test revealed no significant differences between the variation rates of each group. PD = probing depth; CAL = clinical insertion level; BoP = bleeding on probing; PI = plaque index; T = evaluation period; GT = green tea; AH = hyaluronic acid

Parameters/Groups	Periods	Chlorhexidine gel	GT/HA gel
PD (mm)	**T0 – T60**	1.37 ± 0.73	1.34 ± 0.52
Greater PD (mm)	**T0 – T60**	2.31 ± 0.94	2.26 ± 1.04
CAL (mm)	**T0 – T60**	0.81 ± 0.63	0.72 ± 0.49
Greater CAL (mm)	**T0 – T60**	1.47 ± 1.38	1.68 ± 1.10
BoP (%)	**T0 – T60**	39.23 ± 12.57	36.19 ± 14.62
PI (%)	**T0 – T60**	-7.00 ± 8.28	-7.26 ± 10.16

### Microbiological monitoring

**Fig. 5:**
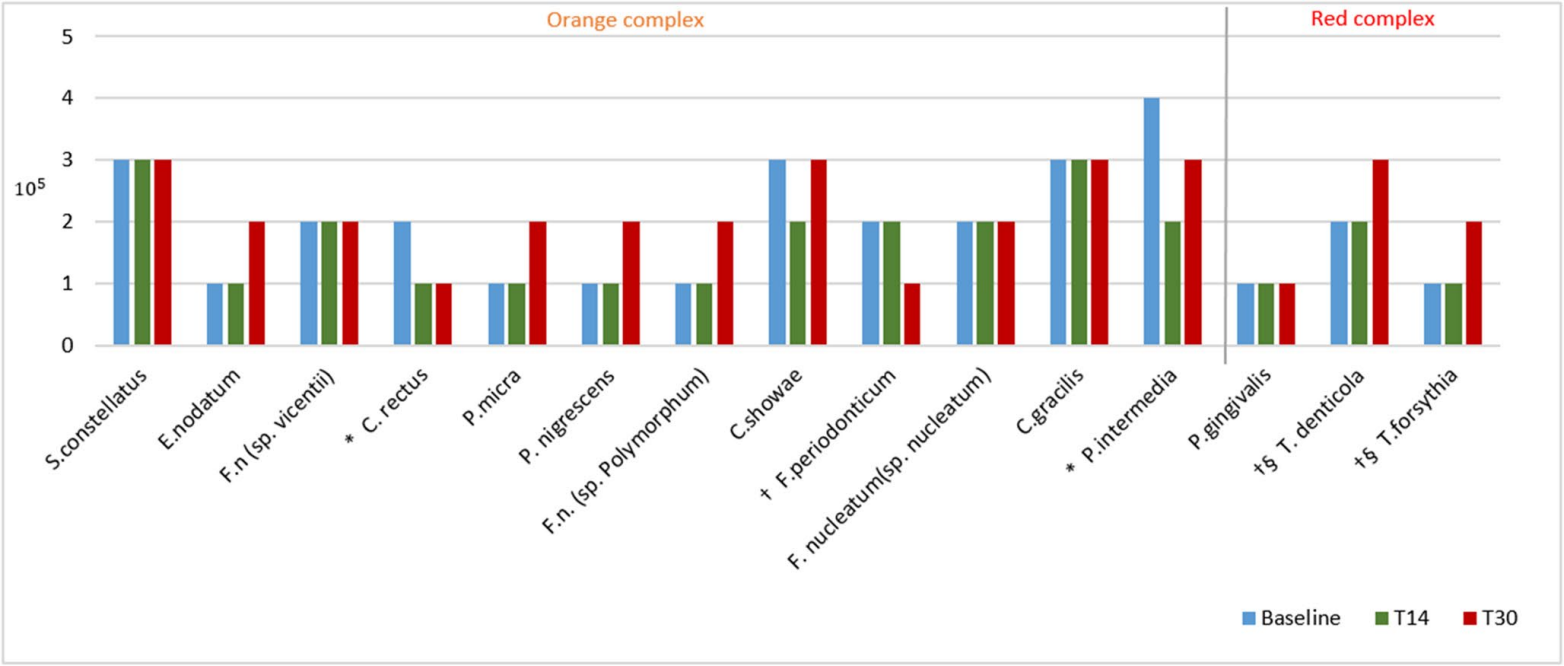
Mean counts of bacterial species from red and orange complexes found in subgingival biofilm samples at baseline and at 14 and 30 postoperative days in the Chlorhexidine group, as well as the results of the within-group comparisons. Significant differences compared with baseline: *14 days; † 30 days. Significant differences compared to 30 days: § 14 days (Wilcoxon test – p < 0.05)

**Fig. 6:**
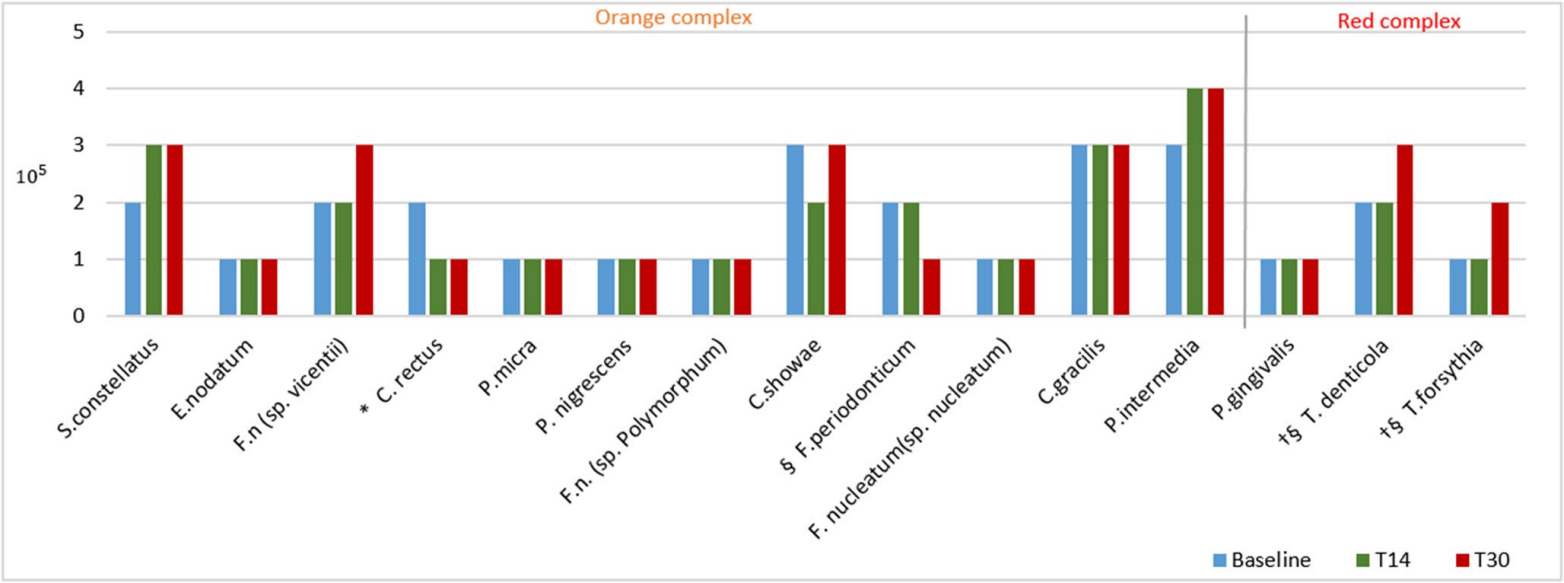
Mean count of bacterial species from red and orange complexes found in subgingival biofilm samples at baseline and at 14 and 30 postoperative days in the GT/HA group, as well as the results of the within-group comparisons. Significant differences compared with baseline: *14 days; † 30 days. Significant differences compared to 30 days: § 14 days (Wilcoxon test – *p* < 0.05)

No significant differences were observed between groups in terms of the mean counts or proportions of any of the tested species at baseline. Both groups had similar results in the microbiological analysis, with few significant differences between them. Differences were observed between the Chlorhexidine and GT/HA groups only in terms of the concentrations of the bacteria *P. intermedia*, *N. mucosa*, *L. buccalis* and *S. gordonii* at 14 days (Mann–Whitney test – *p* < 0.05). The mean bacterial counts for key species from the red and orange complexes across the three time points in the Chlorhexidine and GT/HA groups and the statistically significant differences observed in the mean counts of each group at 14 and 30 days compared with baseline values are shown in Figs. 5 and 6, respectively.

**Fig. 7:**
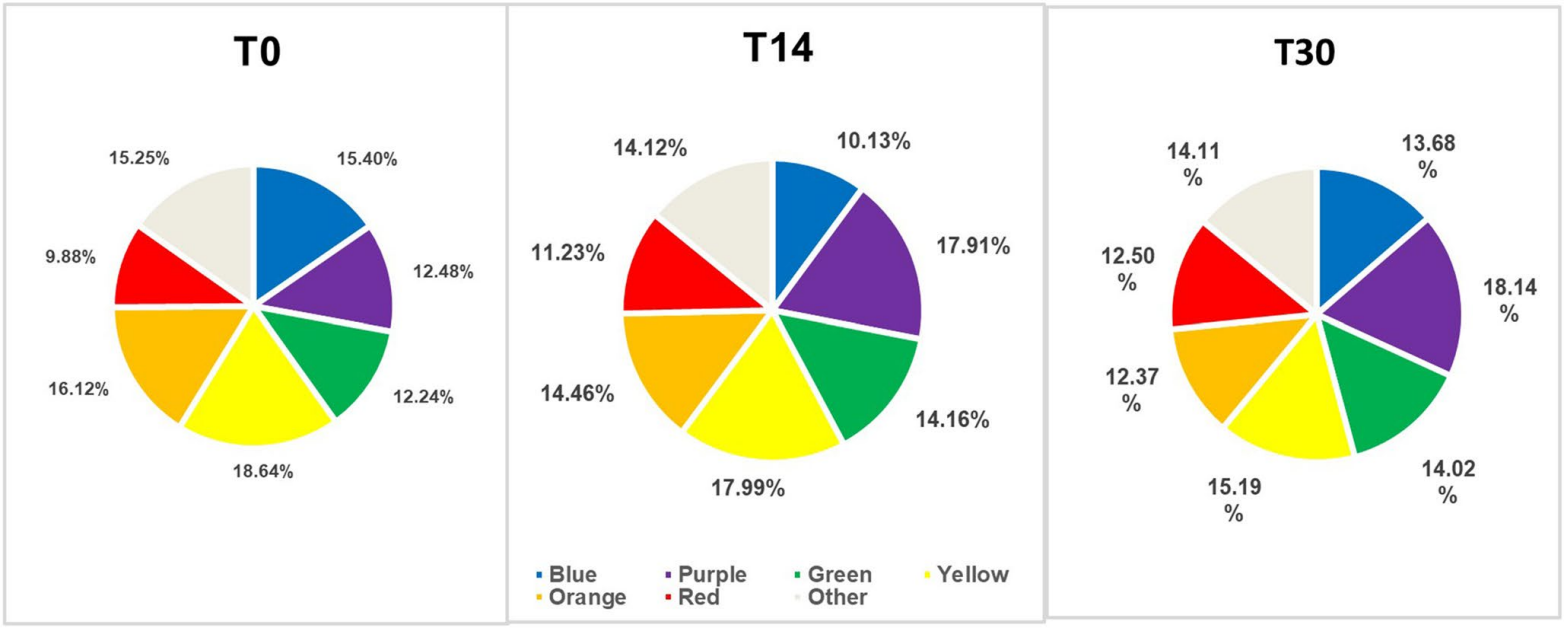
Relative frequencies of the mean percentages of bacterial complexes in different experimental periods in the Chlorhexidine group. No significant differences were found between the different experimental periods (Wilcoxon test). The colors represent different microbial complexes (Socransky et al. 1998)

**Fig. 8:**
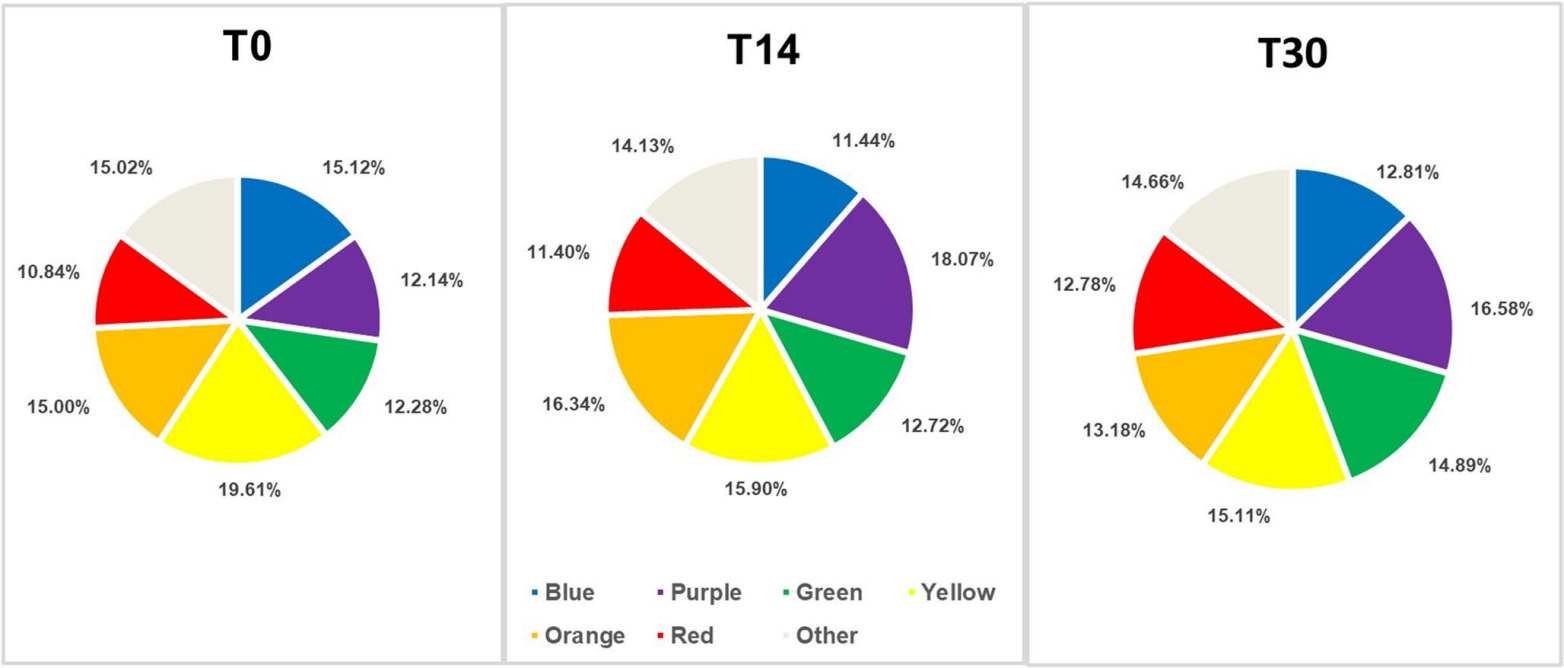
Relative frequencies of the mean percentages of bacterial complexes in different experimental periods in the GT/HA group. No significant differences were found between the different experimental periods (Wilcoxon test). The colors represent different microbial complexes (Socransky et al. 1998)

In both groups, in most bacterial strains, there was a slight or no reduction in the count in the 14-day period in relation to the baseline, and after the use of the gels was discontinued (30 days), the count of bacterial species such as *T. denticola*, *T. forsythia*, *A. actinomycetencomytans*, *C. gingivalis*, *S. noxia* and *P. acnes* (I + II), *S. mitis*, *V. parvula*, and *F. periodonticum* increased (*p* < 0.05) (Figs. 5 and 6). No significant differences were observed in bacterial complexes between the groups during the different experimental periods (Figs. 7 and 8).

### Patient-centered outcomes

#### Postoperative diary and questionnaire

The data collected from the diaries of patients that were given to them on the day of surgery revealed a reduction in pain and analgesic consumption each day after the procedure (Table 2).

**Table 2:** Data collected from the diaries given to patients on the day of surgery, including data on the levels of postoperative pain and the amounts of painkiller taken after surgery. GT = green tea; HA= hyaluronic acid. Different letters represent different levels of differences in each treatment during different evaluation periods (*p* < 0.05; two-way ANOVA complemented by tukey’s test)

Parameters/Groups	Periods	Chlorhexidine gel	GT/HA gel
	T0	3.37 ± 3.21^c^	3.84 ± 2.35^c^
	**T1**	1.47 ± 1.86^b^	1.68 ± 1.57^b^
Pain	**T2**	0.62 ± 1.27^a^	0.99 ± 1.39^a^
	**T3**	0.11 ± 0.49^a^	0.17 ± 0.51^a^
	**T4**	0.10 ± 0.46ª	0.06 ± 0.26^a^
	**T5**	0.08 ± 0.38^a^	0.00 ± 0.00^a^
	**T0**	1.05 ± 0.77^c^	1.26 ± 0.87^b^
Daily painkiller consumption	**T1**	0.52 ± 0.69^b, c^	0.57 ± 0.83^a^
	**T2**	0.31 ± 0.58^a, b^	0.26 ± 0.56^a^
	**T3**	0.00 ± 0.00^a^	0.05 ± 0.22^a^

Additionally, at 7, 14 and 30 days after surgery, patients filled out a questionnaire addressing pain (question 1); sensitivity in the teeth involved in the surgery (question 2); discomfort during eating after surgery (question 3); difficulty during hygiene after surgery (question 4); the presence of bleeding during brushing (question 5); swelling in the surgical area (question 6); the presence of pus in the surgical area (question 7); changes in taste after surgery (question 8); and changes in color in the teeth, restorations or oral tissues (question 9).

The independent variable type of treatment influenced the answers to questions 8 and 9 of the questionnaire. The scores of question 8 (at 7 days) and question 9 (at 7 and 14 days) were higher for users of the chlorhexidine-based gel than for users of the GT/HA-based gel (*p* < 0.05). The independent variable evaluation period had a significant influence on the scores of questions 1 (all treatments) and 8 (only for the chlorhexidine-based gel), with a reduction in these scores as the evaluation period increased (*p* < 0.05) (Table 3).

**Table 3:** Data are presented as the mean and standard deviation of the values ​​obtained by responding to the questionnaires administered to patients in both groups. £ statistically significant difference compared with the GT/HA gel group. Different letters represent different levels of differences in each treatment, varying the evaluation period—*p* < 0.05 two-way ANOVA complemented by the Tukey test

Parameters/Groups	Periods	Chlorhexidine-based gel	GT/HA-based gel
Question 1	**T7**	1.89 ± 2.05^b^	3.00 ± 1.99^b^
	**T14**	0.12 ± 0.52^a^	0.26 ± 1.16^a^
	**T30**	0.00 ± 0.00^a^	0.00 ± 0.00^a^
Question 2	**T7**	2.93 ± 3.35	2.94 ± 2.28
	**T14**	2.88 ± 2.96	2.78 ± 3.23
	**T30**	3.20 ± 2.94	2.31 ± 2.10
Question 3	**T7**	0.81 ± 1.56	1.06 ± 2.20
	**T14**	0.64 ± 1.89	0.84 ± 2.34
	**T30**	0.38 ± 1.24	0.37 ± 1.24
Question 4	**T7**	1.26 ± 2.05	0.89 ± 2.01
	**T14**	0.54 ± 1.53	0.78 ± 2.37
	**T30**	0.50 ± 1.50	0.31 ± 1.15
Question 5	**T7**	0.23 ± 0.71	0.31 ± 0.94
	**T14**	0.49 ± 1.11	0.57 ± 1.56
	**T30**	0.05 ± 0.25	0.36 ± 1.11
Question 6	**T7**	0.38 ± 0.92	0.16 ± 0.53
	**T14**	0.16 ± 0.72	0.15 ± 0.67
	**T30**	0.00 ± 0.00	0.00 ± 0.00
Question 7	**T7**	0.00 ± 0.00	0.00 ± 0.00
	**T14**	0.00 ± 0.00	0.00 ± 0.00
	**T30**	0.00 ± 0.00	0.00 ± 0.00
Question 8	**T7**	2.29 ± 3.08^b£^	0.00 ± 0.00
	**T14**	1.48 ± 3.27^a, b^	0.15 ± 0.69
	**T30**	0.00 ± 0.00^a^	0.00 ± 0.00
Question 9	**T7**	1.02 ± 1.50 ^£^	0.00 ± 0.00
	**T14**	0.76 ± 1.43^£^	0.00 ± 0.00
	**T30**	0.00 ± 0.00	0.00 ± 0.00

#### Oral health impact profile questionnaire

**Fig. 9:**
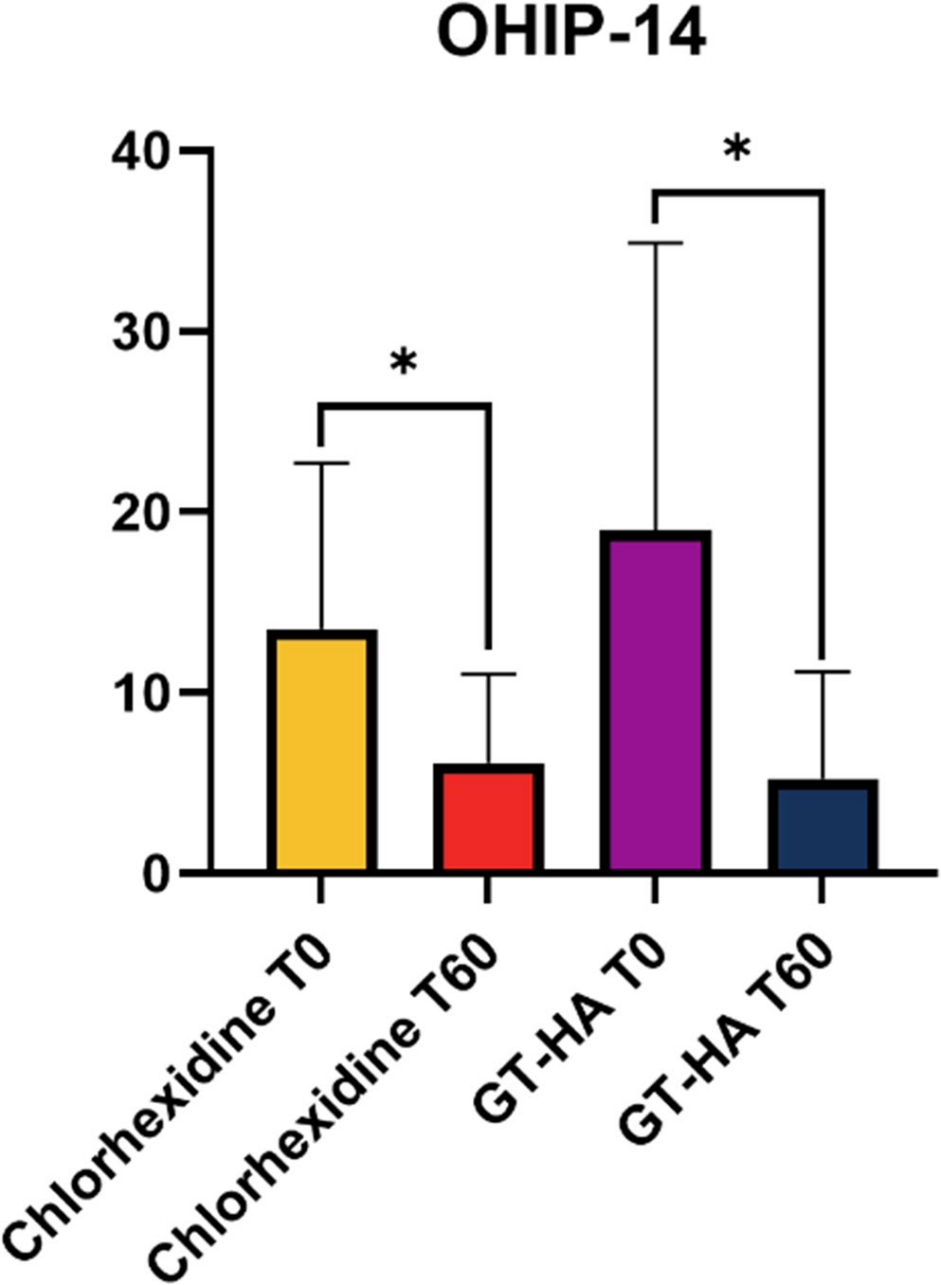
Means and standard deviations of the data obtained from the OHIP-14 questionnaire. * = statistically significant difference (*p* < 0.05 two-way ANOVA complemented by Tukey’s test); GT = green tea; HA = hyaluronic acid

A reduction in the OHIP-14 questionnaire score was observed after surgery, with statistically significant differences between baseline and 30 days after surgery in both groups (*p* < 0.05). In the chlorhexidine group, it decreased from 13.53 ± 9.17 to 6.10 ± 4.90, and in the GT/HA group, it decreased from 18.95 ± 15.92 to 5.26 ± 5.89. (Fig. 9).

## Discussion

This randomized, double-blind clinical trial revealed that the ability of a gel containing green tea extract and hyaluronic acid to control biofilm formation and improve periodontal parameters after surgical intervention for the treatment of periodontitis was non-inferior to that of a chlorhexidine-based gel. For both the clinical parameters analyzed and the impact on oral health-related quality of life (OHIP-14), differences were found between the baseline period and the second evaluation period for both groups, but the type of gel used postoperatively did not significantly differ between the groups. Furthermore, regarding microbiological parameters, the gels had a similar effect on the microbiota of the patients involved in the study.

Currently, the application of this new gel has been evaluated in only one study, in which the gel was applied after third molar extraction surgeries. In an in vitro analysis, Bonatto et al. [21] reported that a gel containing green tea and hyaluronic acid significantly increased the gene expression of AKT, CDKs and VEGF, indicating a positive effect on angiogenesis and human fibroblast proliferation, with a synergistic effect of these two components being observed. When combined, the compounds led to an approximately 100-fold increase in the expression of the AKT gene in relation to that in the control group, revealing the important therapeutic potential of this product for healing and tissue regeneration. Furthermore, clinically, the application of the gel resulted in a reduction in exudate, swelling and secondary interventions, with radiographs showing better alveolar bone density after 90 days. The authors observed a gradual improvement in the clinical aspect of the lesion in both groups over time, but compared with the control group, the test group presented a lower level of alveolar secretion in the lower molars at 7 days after surgery .

As in the study by Bonatto et al. [21], in the present study, patients reported, through postoperative questionnaires, low levels of bleeding during brushing and swelling in the first few days, both in the group in which the GT/HA-based gel was applied and in the chlorhexidine-based gel group. During the 30-day period, no patient reported the presence of these clinical signs. Furthermore, no patient reported signs of infection during the entire postoperative follow-up. The patients in the present study did not report any discomfort when they received the GT/HA-based gel. However, in the postoperative questionnaire (questions 8 and 9), changes in taste and staining of tissues and teeth were reported by patients who used the chlorhexidine-based gel at 7 and 14 days after surgery. At 30 days after surgery, when the use of the gels had been suspended, this complaint no longer existed. These transient adverse effects resulting from the use of chlorhexidine-based products are in agreement with those reported in previous studies on the use of chlorhexidine-based mouthwashes [29–37].

With respect to clinical parameters, the gels exerted similar effects on reducing probing depth and bleeding on probing and increasing the clinical attachment level at 60 days after surgery. These findings agree with the literature, which overall shows positive effects of chlorhexidine [30] and hyaluronic acid-based gels on periodontal parameters after their application.

Mamajiwala et al. [19] verified the influence of a gel containing hyaluronic acid (0.8%) applied to exposed root surfaces during periodontal access surgery for mechanical debridement and reported, at the end of the 12-month study, that in the group in which the gel was used, there was a greater gain in clinical attachment and a greater reduction in probing depth than in the control group, in which no adjuvant treatment was given to the patients. In the study by Fawzy El-Sayed et al. [38], when a hyaluronic acid gel (0.8%) associated with access surgery for scaling was applied, they reported an additional effect of the gel only in relation to the increase in clinical attachment level compared to the placebo group. Notably, in these two studies and in the present study, no gingival collar removal was performed; therefore, the reductions in probing depth and gain in clinical attachment were due only to the surgical procedure and the use of gels. Furthermore, the gels used in each study have different formulations and administration routes, so further studies are needed to verify the effects of the GT/HA-based gel on periodontal parameters after access surgery for scaling in other samples.

Although there are no studies in the literature on the use of green tea-based products for adjuvant therapy after the surgical treatment of periodontitis, some papers have shown that the use of these products with nonsurgical periodontal therapy can enhance its efficacy. A study by Hirasawa et al. [39] highlighted the potential of catechins present in green tea extract, which presented a bactericidal effect against *Porphyromonas gingivalis*, *Prevotella intermedia* and *Prevotella nigrescens* in an in vitro analysis and improved periodontal parameters when combined with mechanical periodontal therapy. These findings regarding the benefits of catechins on clinical parameters after nonsurgical periodontal therapy have also been reported in other studies [15, 40, 41].

The microbiological findings of the present study are similar to those of Duss et al. [30]. In the study by Duss, the authors evaluated the effects of two mouthwashes with different concentrations of chlorhexidine—a control group (0.1%) and a test group (0.05%)—used after periodontal access surgery for scaling. Patients were instructed to use the mouthwash for four weeks after surgery, and subgingival biofilm samples were collected at baseline and at two, four, and twelve weeks after surgery. No differences were found between the groups in the bacterial counts for any species between baseline and the 12th postoperative week. Similarly, in the present study, neither the green tea/hyaluronic acid-based gel nor the chlorhexidine-based gel produced sustained reductions in bacterial counts at 60 days after surgery compared with baseline. However, both formulations induced an initial decrease in bacterial levels at 14 days after surgery, followed by a gradual return to baseline values at 30 days after surgery for some bacterial species, including *A. naeslundii*, *C. rectus*, *P. intermedia*, and *S. mitis* in the chlorhexidine group and *C. rectus*, *S. oralis*, *S. gordonii*, and *S. mitis* in the GT/HA group. Overall, the effects of the gels on the subgingival microbiota were similar.

These results support the notion that chemical adjuncts used in the postoperative phase exert a transient effect on the microbial load, particularly when applied topically rather than systemically. While the long-term microbial recolonization observed here reflects the natural ecological rebound of the subgingival biofilm, the temporary reduction in bacterial counts during the early healing period remains clinically meaningful, as mechanical plaque control is often limited because of postoperative discomfort and surgical restrictions [42, 43].

Consistent with this transient pattern, a progressive increase in the Plaque Index was observed at 60 days in both groups. Recent postoperative studies have similarly shown that the antiplaque effects of chemical agents diminish after their discontinuation, allowing natural biofilm recovery [42]. Reduced patient adherence as healing progresses, particularly in surgically treated posterior areas, and longer intervals between follow-up visits, which limit professional reinforcement of hygiene practices, may further contribute to this increase. Thus, the rise in PI likely reflects ecological biofilm recolonization and a gradual decline in patient compliance [43].

When interpreting microbiological outcomes, methodological considerations should be acknowledged. A potential limitation of the present study is the use of pooled subgingival samples for microbiological analysis. Because periodontitis is a site-specific disease, pooling may attenuate localized microbial variations. However, this approach is justified to carry out checkerboard DNA–DNA hybridization, which requires adequate bacterial DNA to ensure reliable hybridization signals and quantification of multiple species simultaneously [44]. Furthermore, strict standardization procedures were adopted to minimize intraindividual variability: samples were consistently collected from the same preidentified sites (3–4 per patient) with comparable probing depths (5–7 mm) and identical inflammatory status (bleeding on probing). Similar sampling protocols have been successfully applied in randomized clinical trials employing checkerboard DNA–DNA hybridization analysis [45–48]. Therefore, although the pooling strategy may obscure site-specific fluctuations, the methodological precautions taken in this study are expected to minimize this limitation and provide a reproducible and clinically meaningful representation of the patient-level subgingival microbiota.

Another important limitation is the absence of direct or objective assessments of early soft tissue healing. Despite in vitro evidence suggesting that green tea and hyaluronic acid may modulate inflammation and promote cell migration [21], this clinical protocol did not include histological, biomolecular, or standardized photographic assessments of wound repair because of ethical constraints inherent to human studies. Therefore, the clinical improvements observed should not be interpreted as direct evidence of enhanced tissue repair mechanisms. Additionally, the gels were self-administered by patients, and because most surgeries involved posterior teeth, variations in access and visibility may have affected the accuracy of gel placement. Although patients received standardized instructions after surgery and adherence was reinforced at each follow-up visit, the possibility of inconsistent gel distribution must be considered when the magnitude of the antimicrobial effect is interpreted.

Finally, the results of the OHIP-14 questionnaire revealed that the surgeries performed in both groups positively affected the patients’ quality of life. These results are in agreement with those of other studies that reported an improvement in the quality of life of patients after periodontal treatment (surgical and nonsurgical), as measured using the OHIP-14 questionnaire [49–51].

## Conclusion

After the results of this study were analyzed, the following conclusions were drawn:

- The use of a green tea and hyaluronic acid-based gel in postoperative periodontal surgery is not inferior to the use of chlorhexidine-based gel in terms of clinical and microbiological parameters.
- Although long-term changes in the subgingival microbiota were not observed, both gels produced a transient reduction in bacterial counts during the early postoperative phase. This short-term antimicrobial effect is clinically relevant, as it coincides with the critical period in which mechanical plaque control is limited and wound healing is most vulnerable.
- In both groups, there was an improvement in the quality of life related to oral health after surgical therapy, as shown by the decrease in the OHIP-14 questionnaire scores at 30 days after surgery. In addition, compared with the chlorhexidine-based gel, the green tea and hyaluronic acid-based gel presented better results in terms of parameters related to patient well-being, in which there were reports of changes in the color of oral tissues and restorations, as well as in taste.

## Data Availability

The datasets generated and analyzed during the current study are not publicly available because of patient privacy and ethical restrictions but are available from the corresponding author upon reasonable request.
